# A distinct influenza infection signature in the blood transcriptome of patients with severe community-acquired pneumonia

**DOI:** 10.1186/cc11477

**Published:** 2012-08-16

**Authors:** Grant P Parnell, Anthony S McLean, David R Booth, Nicola J Armstrong, Marek Nalos, Stephen J Huang, Jan Manak, Wilson Tang, Oi-Yan Tam, Stanley Chan, Benjamin M Tang

**Affiliations:** 1 Department of Intensive Care Medicine, University of Sydney, Nepean Hospital and Nepean Clinical School, Derby Street, Kingswood, 2747, Australia; 2 Institute for Immunology and Allergy Research, Westmead Millennium Institute, Darcy Road, Westmead, 2145, Australia; 3 Cancer Research Program, The Garvan Institute of Medical Research, 384 Victoria Street, Darlinghurst, 2010, Australia; 4 School of Mathematics and Statistics, University of New South Wales, Kensington, 2052, Australia; 5 Department of Metabolic Care and Gerontology, Charles University Hospital, Sokolska 581, Hradec Králové, 500 00, Czech Republic; 6 Intensive Care Unit, North District Hospital, 9 Po Kin Road, Sheung Shui, Hong Kong; 7 Intensive Care Unit, Pamela Youde Nethersole Eastern Hospital, 3 Lok Man Road, Chai Wan, Hong Kong Island, Hong Kong; 8 Department of Anaesthesia and Intensive Care, Queen Elizabeth Hospital, 30 Gascoigne Road, Kowloon, Hong Kong

## Abstract

**Introduction:**

Diagnosis of severe influenza pneumonia remains challenging because of a lack of correlation between the presence of influenza virus and clinical status. We conducted gene-expression profiling in the whole blood of critically ill patients to identify a gene signature that would allow clinicians to distinguish influenza infection from other causes of severe respiratory failure, such as bacterial pneumonia, and noninfective systemic inflammatory response syndrome.

**Methods:**

Whole-blood samples were collected from critically ill individuals and assayed on Illumina HT-12 gene-expression beadarrays. Differentially expressed genes were determined by linear mixed-model analysis and overrepresented biological pathways determined by using GeneGo MetaCore.

**Results:**

The gene-expression profile of H1N1 influenza A pneumonia was distinctly different from those of bacterial pneumonia and systemic inflammatory response syndrome. The influenza gene-expression profile is characterized by upregulation of genes from cell-cycle regulation, apoptosis, and DNA-damage-response pathways. In contrast, no distinctive gene-expression signature was found in patients with bacterial pneumonia or systemic inflammatory response syndrome. The gene-expression profile of influenza infection persisted through 5 days of follow-up. Furthermore, in patients with primary H1N1 influenza A infection in whom bacterial co-infection subsequently developed, the influenza gene-expression signature remained unaltered, despite the presence of a superimposed bacterial infection.

**Conclusions:**

The whole-blood expression-profiling data indicate that the host response to influenza pneumonia is distinctly different from that caused by bacterial pathogens. This information may speed the identification of the cause of infection in patients presenting with severe respiratory failure, allowing appropriate patient care to be undertaken more rapidly.

## Introduction

The 2009 H1N1 influenza A pandemic reemphasised the important role of respiratory viruses as causes of severe pneumonia. According to World Health Organisation estimates, 450 million cases of pneumonia are recorded every year, and about 4 million people die of this illness [1,2]. In the United States alone, the economic burden of community-acquired pneumonia has been estimated to be more than US$17 billion per annum [3]. The ability to identify patients with viral pneumonia correctly has important patient-management implications, but remains a challenge. Several studies, including [4,5], have shown that the protein biomarkers procalcitonin and C-reactive protein are typically lower in respiratory infections caused by viral as opposed to bacterial infections. These studies, however, were preliminary and consisted of small sample sizes. Attempts also have been made to distinguish clinically between bacterial and influenza pneumonia, by using a combination of variables such as age, mental orientation, temperature, leukocyte count, and chest-radiograph findings [6]. However, the clinical signs and symptoms of bacterial and viral pneumonia can overlap and are often confounded by underlying conditions such as immunosuppression and extrapulmonary complications [7-9]. When these individuals present with community-acquired pneumonia, it is difficult to determine which organism is the causative pathogen (bacterial versus viral).

Assessing the immune response at a gene-expression level may assist in the diagnosis as well as the understanding of the response to pulmonary infections caused by viral compared with bacterial pathogens. We previously showed that in influenza infection, the presence of an abnormal immune response at the gene-expression level is associated with the development of clinical symptoms [10]. Further, we showed that changes in this immune response correlate well with the progression to respiratory failure in infected patients. However, it is not known whether this immune-response signature is specific to influenza infection, or merely a part of a generic host response to infection. Therefore, the aim of this study was to investigate whether a gene-expression signature is present in individuals with severe influenza pneumonia, and whether this immune-response signature is distinct from other conditions that share a similar clinical presentation, such as bacterial pneumonia or systemic inflammation due to noninfectious causes.

## Materials and methods

### Subjects

The study included a total of 39 patients and 18 healthy volunteers. Patients with severe community-acquired pneumonia requiring intensive care unit (ICU) admission were enrolled in the study. Patients with noninfective systemic inflammatory response syndrome (SIRS) also were enrolled (*n *= 12). The study was approved by the Sydney West Area Health Service Human Research Ethics Committee, and informed written consent was obtained from all patients or their relatives. Influenza A H1N1 2009 pneumonia (*n *= 8) was confirmed by using polymerase chain reaction (PCR), and bacterial pneumonia (*n *= 16) by microbiological cultures. Three additional patients were included in the study as a separate group, as they had positive pathology results for both H1N1 influenza A and bacterial infection. Healthy volunteers (*n *= 18) were enrolled in the study as controls. The diagnosis of severe community-acquired pneumonia (caused by bacteria or influenza infection) or SIRS was established at the end of the patient's hospital stay (or after death). SIRS was defined as the presence of at least two of the following four clinical criteria: (a) fever or hypothermia (temperature > 100.4°F (38°C) or < 96.8°F (36°C)); (b) tachycardia (> 90 beats/min), (c) tachypnea (> 20 breaths/min or PaCO_2 _< 4.3 kPa (32 mm Hg)), or the need for mechanical ventilation; (d) an altered white blood cell count of > 12,000 cells/μl, < 4,000 cells/μl, or the presence of > 10% band forms. Pneumonia was defined as a microbiologically confirmed infection of the lungs, resulting in the patient fulfilling the SIRS criteria. The diagnosis was ascertained by using all the information available in the patient's medical records. This information included microbiology reports, PCR results, image studies (for example, computed tomography scans), surgical findings, tissue histopathology reports, and response to antibiotics. The physician who determined the reference diagnosis was blind to the results of the microarray analysis. Whole-blood samples were drawn from all subjects. The first sample from each patient was collected within the initial 24 hours of admission to the ICU, henceforth referred to as day 1. Patients were monitored for up to 5 days to assess their longitudinal gene-expression profiles. Sampling was performed only on days 1 and 5 in the healthy control cohort, as we did not expect significant changes in gene-expression profiles from day to day. For critically ill individuals, clinical characteristics, including APACHE II (Acute Physiology And Chronic Health Evaluation score II [11]), age, gender, comorbidities, length of ICU stay, and mortality, were collected.

### Gene-expression profiling

Whole-blood samples were collected into PAXgene tubes and immediately stored at -20°C. RNA extraction was performed by using the standard protocol (PAXgene Blood RNA kit, Qiagen, Hilden, Germany). RNA quality was analyzed by using Agilent 2100 Bioanalyser (Agilent Technologies, Santa Clara, CA, USA), and all samples obtained an RNA integrity number of greater than 6.5, indicating high sample quality. Extracted RNA was stored at minus 80°C until expression profiling, by using Illumina Sentrix HT-12_v3_BeadChip arrays (Illumina, San Diego, CA, USA). Sample amplification and labeling was carried out on 200 ng of total RNA by using an Illumina TotalPrep Amplification kit (Ambion, Austin, TX, USA). Amplified complementary RNA was assessed by using the Agilent 2100 Bioanalyser, to ensure satisfactory amplification. The samples were then immediately hybridized onto HT-12_v3_BeadChips; 750 ng of each sample was loaded onto the arrays. The hybridization and washing procedure was identical for each set of arrays processed. To minimize experimental artefacts, all of the RNA extraction, sample amplification and labeling, hybridization and washing, and scanning procedures were carried out by the same operator, at the same time of day. After raw-data processing and normalization, no significant batch effects were identified. Therefore, no additional adjustment of the microarray data was required. The microarray data discussed here have been deposited in the NCBI Gene Expression Omnibus [12] and are accessible through GEO Series accession number GSE40012 [13].

### Bioinformatic workflow

Raw data obtained by scanning of the microarray slides were processed by using Illumina GenomeStudio V2010.3. Each probe on the array was passed through a filter requiring a detection *P *value of < 0.0050 in at least one sample to be included in any further analyses. Of the 48,804 probes present on the Illumina HT 12 array, 24,840 probes (henceforth referred to as genes) passed this criterion. Genes that passed the filtering were loaded into BRB ArrayTools [14], in which quantile normalization and log transformation of the data were applied. Validation of the microarray experiment was performed by measuring the expression relative to GAPDH for a subset of genes, by using qRT-PCR. The R^2 ^values obtained when comparing qRT-PCR and microarray relative fold-changes ranged from 0.67 to 0.83, indicating strong concordance between the two platforms.

Normalized and log-transformed data were imported into R (v2.12). Genes with low variance across all samples, defined to be less than the median, were removed from the dataset. This left 12,420 genes to be used for statistical analyses. Each patient phenotype was compared with the healthy control cohort by fitting a linear mixed model to each gene by using the R library lme4. Patient phenotype, day of ICU stay, gender, age, patient ID, and APACHEII score (disease severity) were all included in the model as independent variables. This allowed the selection of genes significant for phenotype after accounting for each of the other terms in the model. *P *values were adjusted for multiple testing by using the Benjamini and Hochberg False Discovery Rate (FDR) method [15] (R library multitest). An FDR of 5% was used as the cut-off for genes deemed to be differentially expressed between the two classes.

Differentially expressed gene lists were uploaded into GeneGo Metacore (St. Joseph, MI, USA), an integrated software suite for functional analysis of gene-expression data. With GeneGo MetaCore, biological pathway analysis was performed on each gene list. Pathway analysis involved matching a list of prespecified genes onto canonic pathways or networks and calculating the statistical relevance of the matches found. An FDR of 5% was used as the cut-off to determine whether a pathway was statistically overrepresented in the gene list.

To identify the particular immune cell subsets contributing to genes dysregulated in response to influenza and bacterial pneumonia, we performed a process referred to as immune cell deconvolution. First, the top 100 genes sorted by statistical significance were determined for genes upregulated in H1N1 influenza A pneumonia and also for genes upregulated in bacterial pneumonia. Each of the genes in these lists was subsequently searched for by using the ImmGen database [16] to assess their immune cell subset-specific expression. A gene was said to tag a particular immune-cell type if it was overexpressed in fewer than four different immune-cell types. A Fisher Exact test was used to determine whether any significant difference existed in proportion of immune cell-tagging genes in genes expressed higher in H1N1 influenza A pneumonia compared with genes expressed higher in bacterial pneumonia patients.

To test for the enrichment of a list of known interferon-stimulated genes [17] in the influenza and bacterial pneumonia groups, a technique called Gene Set Enrichment Analysis was performed [18,19]. Gene Set Enrichment Analysis was performed on gene lists created by ranking genes by the *P *value generated for phenotype in the linear mixed-model analyses from most significant to least significant.

To quantify further the differences in gene-expression pattern of the H1N1 influenza A and bacterial pneumonia samples on day 1 of admission to ICU, a Support Vector Machines (SVM) class predictor was built [20]. A *P *value of 1E-5 was chosen as the optimal threshold for deciding the genes to be included in the class predictor for distinguishing day 1 samples of H1N1 influenza A pneumonia and bacterial pneumonia. A more-stringent *P *value threshold resulted in a reduction of the number of genes used in the class predictor; however, this also resulted in a reduction of the mean percentage of correct classification. See Additional file 1, Table S1, for the *P*-value thresholds tested and the resulting number of genes used, as well as the mean percentage of correct classification for each class predictor. Performance of the class predictor was assessed in the training dataset by using the leave-one-out cross-validation method [21] and was also assessed in two independent datasets [22,23]. The first independent dataset, published by Ramilo *et al*. [22], consists of peripheral blood mononuclear cell samples of bacterial sepsis and influenza A and B patients. The second dataset, published by Bermejo-Martin *et al*. [23], consists of PAXgene whole-blood samples from individuals with severe H1N1 influenza A pneumonia, compared with healthy controls. By using the weightings and the threshold determined in the training set, the SVM integer was plotted for each of the samples in the two independent validation cohorts. The SVM integer was calculated by multiplying the predetermined weight for each gene by its corresponding expression level, and adding these values for each of the genes in the class predictor. Biological pathway analysis and immune cell deconvolution was carried out on the gene-list used to build the class predictor.

A cluster analysis was performed to visualize the difference in expression profile between samples collected from patients with concurrent bacterial and H1N1 influenza A infections as opposed to SIRS, bacterial pneumonia, or H1N1 influenza A pneumonia patients. A clustering dendrogram was generated by using the genes used to build the SVM class predictor, by using euclidean distance and average linkage metrics. The dendrogram included day 1 samples of the bacterial and H1N1 influenza A groups as well as day 1 samples for three patients with concurrent bacterial and H1N1 influenza A infections. Day 1 samples from the noninfectious SIRS cohort also were included.

## Results

Characteristics of each of the patient groups are summarized in Table 1. For each of the bacterial pneumonia patients, the pathogen responsible for infection and the specimen from which the result was obtained is listed in Additional file 1, Table S2. No difference in the severity of illness (as measured by APACHE II scores) was found for patients in the bacterial pneumonia compared with the H1N1 influenza A pneumonia group (*P *= 0.82). The mean age of bacterial pneumonia patients was higher than that of the influenza A patients (*P *= 0.00040). We therefore incorporated age as a covariate in the linear mixed-model analysis. All results reported henceforth have accounted for the difference in age between groups.

**Table 1:** Characteristics of the individuals included in the study

	H1N1 influenza A	Bacterial pneumonia	SIRS	Mixed influenza A/bacterial pneumonia	Healthy control
Demographics					
Number in group	8	16	12	3	18
Age (years)	34 ± 12	62 ± 13	61 ± 16	39 ± 13	43 ± 16
Male/Female	3/5	7/9	10/2	1/2	6/12
Comorbidities (%)					
Hypertension	12.5	31.3	58.3	0	16.6
Heart disease	0	37.5	33.3	33.3	0
Diabetes	12.5	12.5	25.0	0	0
COPD	25	37.5	16.7	0	0
Cancer	0	12.5	0	0	0
Trauma	0	0	8.3	0	0
Recent surgery (last 7 days)	0	6.25	33.3	33.3	0
Severity of disease					
Mortality (%)	0	31.3	0	0	NA
APACHE II	15 ± 3.8	18 ± 6.6	16 ± 4.5	18 ± 5.6	NA
Treatment (%)					
Mechanical ventilation	100	93.8	75	100	NA
Renal dialysis	12.5	6.3	8.3	33.3	NA
Vasopressor therapy	62.5	56.3	16.7	66.6	NA

APACHE II, Acute Physiology And Chronic Health Evaluation score [11]; COPD, chronic obstructive pulmonary disease; SIRS, systemic inflammatory response syndrome. Plus-minus values are median ± standard deviation.

The linear mixed-model analysis showed that changes in levels of gene expression were determined by patient phenotype (H1N1 influenza A, bacteria, or SIRS). Other variables, such as disease severity, day of ICU stay, and patient age, were not associated with any change in gene-expression levels. With the exception of Y-linked genes *RPS4Y1, JARID1D*, *EIF1AY, UTY*, and *RPS4Y2*, patient gender was not found to influence gene-expression levels. Each phenotype was associated with significant changes in gene expression in a large number of genes, as summarized in Table 2.

**Table 2:** Number of genes up- and downregulated for each patient phenotype, compared with healthy controls

Comparison (*n *versus *n*)	Up	Down	FDR
H1N1 influenza A versus healthy control (8 versus 18)	3,244	2,902	5%
Bacteria versus healthy control (16 versus 18)	2,434	2,661	5%
SIRS versus healthy control (12 versus 18)	2,775	2,973	5%

**Figure 1:**
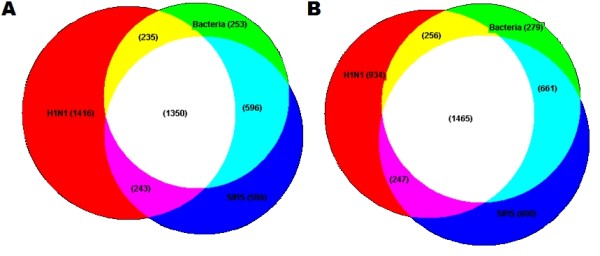
**Overlap of differentially expressed genes in H1N1 influenza A pneumonia, bacterial pneumonia, and noninfective systemic inflammatory response syndrome**. Venn diagrams for genes upregulated **(A) **and genes downregulated **(B) **compared with healthy controls, at 5% false-discovery rate. H1N1 influenza A pneumonia (H1N1), bacterial pneumonia (Bacteria), noninfective systemic inflammatory response syndrome (SIRS).

Venn diagrams reveal overlaps in the lists of upregulated and downregulated genes compared with healthy controls for the three patient phenotypes (Figure 1A, B). At 5% FDR, 1,350 genes were upregulated compared with healthy controls in all three patient phenotypes. Biological pathways overrepresented in these genes included apoptosis (p = 4.4E-8), immune system response (*P *= 4.3E-6), DNA-damage response (*P *= 1.4E-5), and inflammatory response (*P *= 6.8E-5).

**Figure 2:**
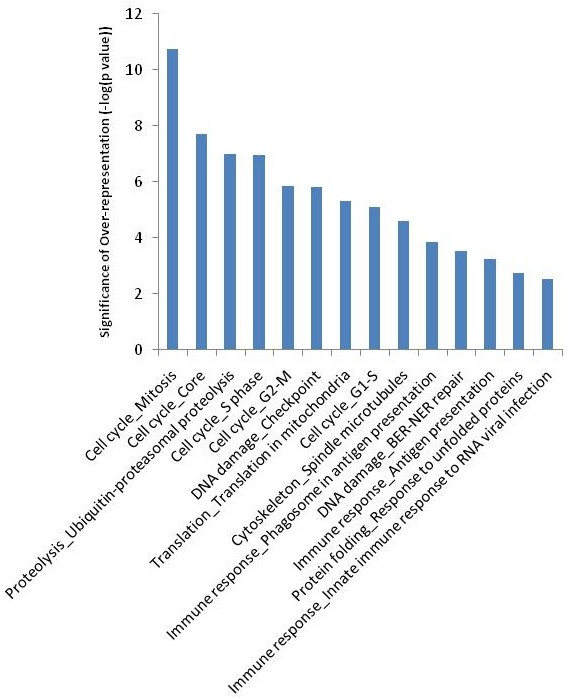
**The top-ranking biological pathways in genes upregulated in H1N1 influenza A infection, ordered by statistical significance (with cell cycle being the most significant among the top 10 pathways)**.

A distinct gene-expression profile was found for the H1N1 influenza A group. This gene-expression profile is found predominantly in the upregulated genes (Figure 1A). Biological pathway analysis of the 1,416 genes uniquely upregulated in H1N1 influenza A infection revealed overrepresentation of pathways related to the cell cycle and its regulation (p = 4.2E-20), DNA-damage response (*P *= 4.2E-9), apoptosis (*P *= 1.3E-4), and protein degradation (*P *= 4.1E-4). Figure 2 lists the top overrepresented biological pathways in the order of statistical significance.

In contrast to influenza A infection, a gene-expression signature was not found in bacterial pneumonia. The genes uniquely upregulated in response to bacterial pneumonia (*n *= 253) were not overrepresented in any biological pathway or network ontology, implying a generic inflammatory and immune response, but no specific response to bacterial infection.

A larger number of genes were upregulated in SIRS (586 genes). Further analysis showed that they were overrepresented in multiple biological pathway and network ontologies, including inflammatory response (*P *= 6.3E-6), cell differentiation (*P *= 1.6E-5), angiogenesis (*P *= 1.1E-4), and immune system response (*P *= 2.6E-4). This is consistent with the known biology of SIRS, which is a nonspecific host response to a variety of stresses, including trauma, surgery, and infection.

**Figure 3:**
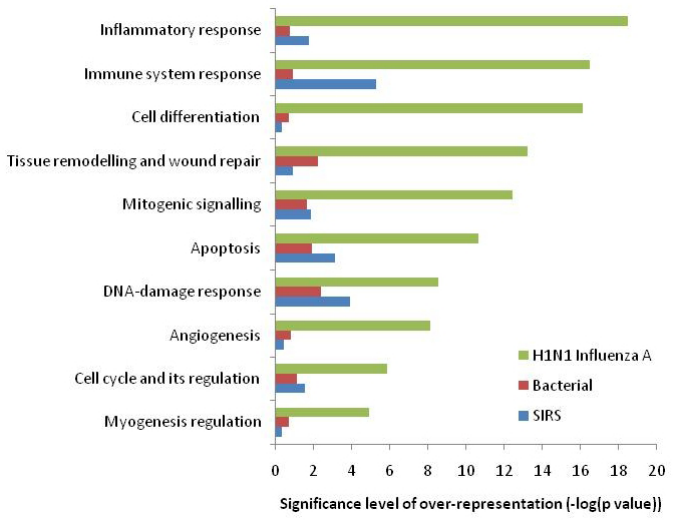
**Representation of biological pathway ontologies in the downregulated genes at 5% false discovery rate (FDR) for H1N1 influenza A, bacterial pneumonia, and systemic inflammatory response syndrome (SIRS), compared with healthy controls**.

A large number of genes were downregulated in H1N1 influenza A infection, bacterial infection, and SIRS groups (Figure 1B). Biological pathway analysis of the downregulated genes was performed for each of the three patient phenotypes (Figure 3). In the H1N1 influenza A group (934 unique genes), many genes were overrepresented in inflammatory-response and immune system-response pathways. Further interrogation into the immune-response pathways showed that activation and signaling pathways of interleukins (IL-8, IL-2, IL-15, IL-6, IL-10, IL-7, IL-3, IL-13, IL-17, and IL-23) were heavily overrepresented in the downregulated gene list. This suggests a significant degree of immunosuppression in severe H1N1 influenza A infection. In contrast, the degree of downregulation in biological pathways was considerably less in both the bacterial-infection and the SIRS groups (Figure 3).

Pathway analysis of the direct comparison between the H1N1 influenza A and bacterial groups revealed a consistent picture, with 671 genes upregulated in H1N1 influenza A compared with bacterial (by using linear mixed model, 5% FDR) showing remarkable overrepresentation in the cell cycle and its regulation ontology (*P *= 2.9E-20). The DNA-damage response was also highly enriched in this list of genes (*P *= 6.9E-10). No such overrepresentation was seen for cell-cycle pathways in the 78 genes expressed at higher levels in the bacterial infection group (*P *= 0.35). The biological pathways overrepresented by these 78 genes include immune and inflammatory responses. However, these immune/inflammatory genes are also upregulated in SIRS and are therefore not specific to bacterial pneumonia.

**Figure 4:**
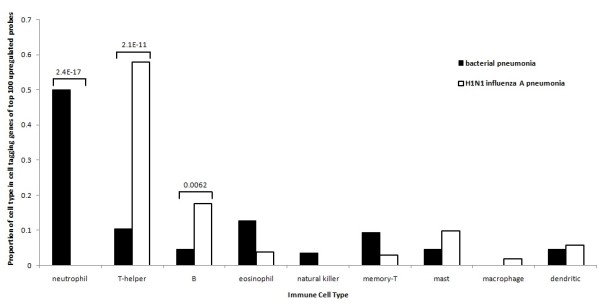
**Immune cell deconvolution of the top 100 upregulated genes for bacterial pneumonia and H1N1 influenza A pneumonia, compared with healthy controls**. Fisher Exact test two-tailed *P *values are given for cell types with significantly different proportions between the two groups.

**Figure 5:**
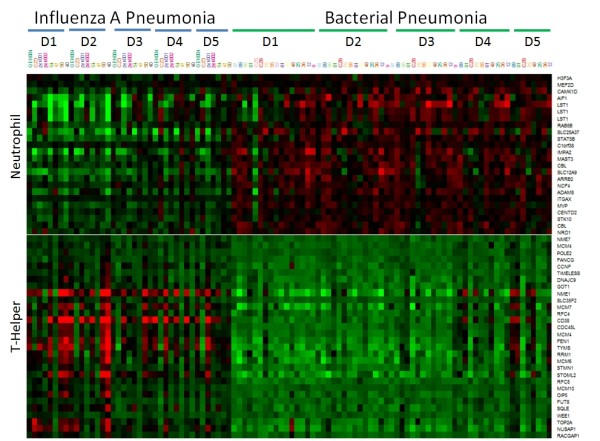
**Expression of neutrophil and T-helper cell-specific genes across 5 days for H1N1 influenza A pneumonia and bacterial pneumonia patients**. Intensity of red corresponds to level of upregulation, whereas intensity of green refers to level of downregulation.

The immune cell subsets that gave rise to most of the gene-expression signals outlined earlier are shown in Figure 4, as revealed by immune cell deconvolution. Far more neutrophil-tagging genes were upregulated in the bacterial group compared with the H1N1 influenza A pneumonia (*P *= 2.4E-17). Conversely, a greater representation of T-helper cell-tagging genes was found in the top 100 upregulated genes for H1N1 influenza A pneumonia (*P *= 2.1E-11). In addition, B-cell genes were significantly overrepresented in the H1N1 influenza A pneumonia group compared with the bacterial group (*P *= 0.0062). These findings are consistent with the known biology of infection, in which bacterial infection is driven by a neutrophil-dominant response, and viral infection is driven by a lymphocyte-dominant response. Across the 5 days of patient follow-up, the expression level of T-helper cell-tagging genes is consistently higher in H1N1 influenza A, whereas the expression level of the neutrophil-tagging genes is consistently higher in the bacterial group, as shown in Figure 5.

A group of genes well known to be associated with viral infection, referred to as interferon-stimulated genes, were highly represented in the H1N1 influenza A gene signature. With Gene Set Enrichment Analysis, the interferon-stimulated genes were shown to be significantly enriched in the genes overexpressed in H1N1 influenza A pneumonia, compared with healthy controls (FDR = 0.0010). In contrast, even at a 5% FDR, no significance was observed for interferon-stimulated genes among genes overexpressed in bacterial pneumonia, compared with healthy controls (FDR = 0.080). We repeated the analysis by directly comparing the bacterial and H1N1 influenza A groups. Again, a highly significant enrichment of the interferon-stimulated genes was noted in genes overexpressed in the H1N1 influenza A group (FDR = 0.0010) but not for genes overexpressed in the bacterial group (FDR = 0.97).

**Figure 6:**
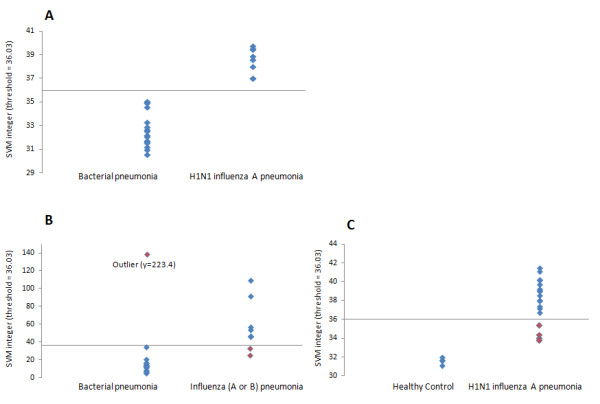
**The Support Vector Machines (SVM) class-prediction integer in training and validation datasets**. The x-axis corresponds to the threshold of 36.03, with all samples falling above the line predicted as belonging to an individual with influenza infection. Correctly predicted samples are shaded blue; incorrectly predicted samples are shaded red.

Because the H1N1 influenza A infection group displayed a gene-expression profile distinctively different from that of bacterial infection, we explored the potential of using the gene-expression profile to diagnose H1N1 influenza A infection. By using an SVM algorithm, we found a 29-gene class predictor to be highly accurate in discriminating H1N1 influenza A infection from bacterial pneumonia (Figure 6). This ability to discriminate between bacterial and viral infection was consistent across the 5 days of patient follow-up (see Additional file 1, Figures S1 and S2). When this class predictor was tested on two independent datasets, it was shown to provide clear separation of both H1N1 influenza A pneumonia patients from a healthy control cohort, and bacterial pneumonia patients from a cohort of patients containing both influenza A- and influenza B-infected individuals. These results support the robustness of the class predictor, as clear separation was observed in independent datasets generated by using different microarray platforms and normalization methods.

Surprisingly low overlap is found when comparing the 29-gene class predictor presented in this study with the class predictors presented in previous studies by Zaas *et al*. [24] (30 genes), and Ramilo *et al*. [22] (35 genes). Only five genes are present in more than one of the three-gene signature lists: *IFI44, LY6E, MX1, OAS1*, and *IFI27 *(see Additional file 1, Table S3). Notably, each of these five genes is a well-established interferon-inducible gene.

Further analysis of the 29-gene signature showed overrepresentation in biological pathways related to the cell cycle and its regulation (*P *= 2.1E-4). Specific cell-cycle pathways overrepresented were transition and termination of DNA replication (*P *= 7.1E-4) and start of DNA replication in early S phase (*P *= 9.3E-4). No other pathway ontology was significantly overrepresented in the 29-gene signature. Immune cell deconvolution of the 29-gene signature revealed that 14 of the 29 genes were predominantly expressed in T-helper cells. This finding suggests that the 29-gene signature reflects the T-cell response during influenza infection.

**Figure 7:**
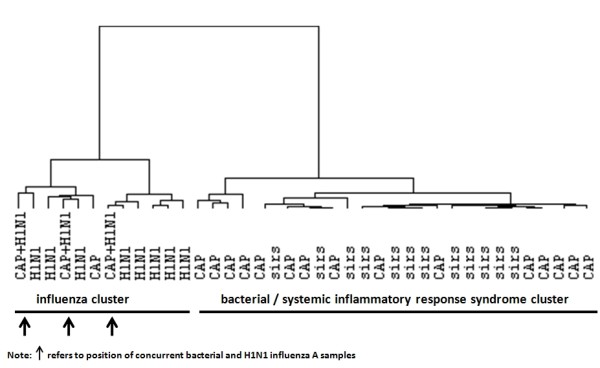
**Dendrogram for clustering bacterial (CAP), H1N1 influenza A (H1N1), systemic inflammatory response syndrome (SIRS), and concurrent bacterial and H1N1 influenza A infection (CAP+H1N1) patients for the 29-gene signature by using Euclidean distance and average linkage (all samples were obtained on day 1)**.

The diagnostic performance of the 29-gene signature to identify viral infection remained high even for patients with concurrent bacterial coinfection. We performed an analysis on blood samples of three patients who had both H1N1 influenza A infection and superimposed bacterial infection. Figure 7 shows the cluster analysis after these new samples were incorporated into our original dataset. With the 29-gene signature, all the H1N1 influenza A samples fell into the first cluster, whereas the bacterial or SIRS samples were grouped in a second cluster. Importantly, all three patients with viral and bacterial coinfection were in the H1N1 influenza A group. This suggests that the 29-gene viral signature is not affected by the presence of a bacterial coinfection. One of these three patients had an additional sample collected on day 13. At this point, the H1N1 influenza A pneumonia had been resolved; however, the bacterial infection remained. We note with interest that the day-13 sample was more similar to the bacterial infection cohort in its gene-expression profile. The repeated cluster analysis on day 13 showed that this patient had migrated to the bacterial and SIRS cluster (data not shown).

## Discussion

Our whole-blood expression profiling data indicate that H1N1 influenza A pneumonia has an immune response detectable at a gene-expression level. This immune response persists beyond the first 24 hours of admission to the ICU and was present throughout 5 days of follow-up. In addition, the H1N1 influenza A signature was highly consistent, as it remained detectable in a subset of patients with concurrent bacterial infection. Furthermore, this signature is highly specific to viral pneumonia (influenza A), because it is distinctively different from the gene-expression profile of bacterial pneumonia, or any conditions that may mimic an inflammatory host response. Our data therefore provide proof-of-concept evidence that gene-expression profiling may identify the etiology of acute pulmonary infection in critically ill patients, allowing more-specific patient care.

Our study addresses an important issue in the current diagnosis of influenza infection. The current diagnosis in critically ill patients is difficult because of a lack of correlation between influenza virus antigen test and clinical status. For example, many influenza-infected individuals test negative for influenza virus [25]. Our study showed that the key to diagnosis is the presence of an abnormal immune response associated with influenza virus. This makes biological sense because it is the abnormal immune response that determines the progression to a more-severe illness, or sometimes, death. The 29-gene signature reflects the virus-specific host immune response. This allows influenza infection to be diagnosed correctly, independent of the result of the viral antigen test. Furthermore, the persistence of the 29-gene signature over time makes it possible to diagnose viral pneumonia for at least 5 days after ICU admission. Many critically ill patients present late, often with impending respiratory failure. By this stage, the viral shedding is minimal, and the pick-up rate for viral antigen testing is low. Another useful application of our gene-expression signature will be to assist the diagnosis in patients with bacterial coinfection. During the influenza season, many patients with bacterial pneumonia also test positive for the influenza virus, making it difficult to ascertain the etiology of the infection. The 29-gene viral gene signature has the potential to resolve diagnostic uncertainty in this situation by directly demonstrating the presence of a virus-specific immune response. These results warrant further exploration in a future diagnostic study in which the gene-expression signature can be validated in a large independent patient cohort.

Deconvolution of whole-blood gene-expression data, a novel method developed to gain insight into immune cell-subset gene expression [10,26,27], revealed a strong representation of T-helper cell-expressed genes upregulated in the whole-blood gene signature for H1N1 influenza A. Previous findings reported that H1N1 influenza A infection was characterized by a T-helper cell response, in particular type 1 and type 17 T-helper cells [28]. Conversely, a lack of T-helper cell response was noted in the bacterial pneumonia gene signature, which was characterized by large representation of neutrophil-expressed genes. This finding reinforces our finding that our 29-gene viral signature reflects the actual immune response of the host during influenza infection.

Our study also revealed two surprising new findings. First, immune and inflammatory pathways genes have been traditionally thought of as the main determinants of host response to influenza infection. Our findings show that genes linked to the cell cycle and its regulation were the main determinants of the host response in influenza infection, whereas most immune and inflammatory genes were downregulated. This downregulation points more toward a state of immune suppression, particularly so for many interleukin-receptor and signaling pathways. This finding has potential diagnostic implications. For decades, inflammatory or immune response genes and proteins have been investigated for their utility as diagnostic markers for bacterial and viral infection. However, many of these markers have failed because of the lack of specificity (for example, the marker is expressed in both viral and bacterial infection). Our results suggest that cell cycle-related genes may provide alternative candidates as diagnostic markers.

The second surprising finding is that our study failed to identify an immune response specific to bacterial pneumonia. A small number of genes were dysregulated uniquely in the bacterial infection group. However, analysis of these genes revealed that no particular biological pathways or networks were significantly overrepresented. The gene-expression pattern of the bacterial group was most similar to the SIRS cohort, as demonstrated by the large overlap of genes between these two groups in the Venn diagrams (Figure 1) and in the cluster analysis (Figure 7). Further evidence supporting a lack of a unique immune response to bacterial pneumonia was mounted when the SIRS and bacterial pneumonia cohorts were compared directly, by using the linear mixed model. No genes were significantly differently expressed between these two phenotypes at 5% FDR (data not shown). The lack of a bacteria-specific gene signature contrasts sharply with the discovery of the 29-gene virus-specific signature.

In this study, we focused on one specific cause of viral pneumonia, pandemic H1N1 influenza A. From the results we have presented, we are unable to conclude whether the gene-expression signature we have identified is specific to pandemic H1N1 influenza A viral infection, specific to all subtypes of influenza, or a generic response to respiratory viruses (for example, rhinovirus, respiratory syncytial virus, influenza A and B). This was addressed to a small extent: one of the independent test datasets we used contained both influenza A- and B-infected individuals. In this dataset, all of the influenza-infected samples exhibited a similar gene-expression signature, as calculated by the SVM integer (Figure 6B). Attempts have been made by others to address this question by including multiple respiratory virus types [24], and their results point toward a relatively conserved nature of the host response to viral infection. A signature that distinguishes a response to a viral opposed to a bacterial infection would be useful in the clinical management of pneumonia patients. Confounding variables such as effect of therapeutic interventions, including medications, should be addressed in future studies with a larger sample size; however, this is outside the scope of this study.

## Conclusions

We have identified a T-cell-dominant gene-expression signature that is associated with the host response to severe influenza pneumonia. This signature provides an insight into the pathophysiology of influenza and may serve as an alternative diagnostic approach to assist in the management of severe community-acquired pneumonia. The validity of such an approach warrants further study in a large independent patient cohort.

## Key messages

• The whole-blood gene-expression profile of H1N1 influenza A was distinctly different from bacterial pneumonia and systemic inflammatory response syndrome.

• Increased expression levels of genes linked to the cell cycle and its regulation were the main determinant of the host response in influenza infection, whereas most immune and inflammatory genes were downregulated.

• Deconvolution of the whole-blood gene-expression data revealed a strong representation of T-helper cell-expressed genes upregulated in the whole-blood gene signature for severe H1N1 influenza.

## Abbreviations

APACHE II: Acute Physiology And Chronic Health Evaluation score II; DNA: deoxyribonucleic acid; FDR: Benjamini and Hochberg false discovery rate; ICU: intensive care unit; PCR: polymerase chain reaction; qRT-PCR: quantitative reverse transcriptase polymerase chain reaction; RNA: ribonucleic acid; SIRS: systemic inflammatory response syndrome; SVM: Support Vector Machine.

## Competing interests

The authors declare that they have no competing interests.

## Authors' contributions

BT, SH, AM, and GP conceived and designed the experiments. GP performed the experiments. GP, DB, and NA analyzed the data. GP wrote the manuscript. GP, BT, and DB interpreted the data. JM, WT, OT, SC, MN, BT, GP, and SH undertook patient recruitment and clinical data collection. BT, SH, MN, NA, and AM contributed to the revision of the manuscript. All authors read and approved the manuscript for publication.

## Supplementary Material

Additional file 1 **Supplementary results**. This file contains three supplementary tables and two supplementary figures, as cited in the main text. Click here for file
