# Supplementary material for: A distinct influenza infection signature in the blood transcriptome of patients with severe community-acquired pneumonia
Source: Crit Care. 2012 Aug 16;16(4):R157. doi: 10.1186/cc11477 (PMC3580747; doi:10.1186/cc11477)
Supplement: Additional file 1 — Supplementary results. This file contains three supplementary tables and two supplementary figures, as cited in the main text. [file cc11477-S1.DOC]

**Table S1. Optimisation of the p-value threshold for the H1N1 influenza A pneumonia and bacterial pneumonia class predictor**

| p-value threshold | Number of genes in class predictor | Mean percentage of correct classification |
| --- | --- | --- |
| 5e-5 | 79 | 79 |
| 1e-5 | 29 | 83 |
| 5e-6 | 19 | 79 |
| 1e-6 | 10 | 78 |
| 5e-7 | 5 | 53 |

**Table S2**: Causal Pathogens for bacterial Pneumonia Patients

| Patient ID | Pathogen | Specimen |
| --- | --- | --- |
| 12 | streptococcus | Bronchoalveolar lavage, urine |
| 25 | Staphylococcus aureus | Sputum |
| 36 | Coagulase negative Staphylococcus | Blood |
| 48 | Streptococcus pneumoniae | Blood |
| 75 | Mycoplasma pneumoniae | Bronchoalveolar lavage |
| 81 | Staphylococcus aureus | Blood |
| 89 | Streptococcus pyogenes | Blood |
| 9 | Haemophilus influenzae | Sputum |
| 42 | Chlamydophila pneumoniae | Blood |
| 52 | Klebsiella pneumoniae | Bronchoalveolar lavage |
| 55 | mixed pyogenic bacteria | Pleural fluid |
| 61 | Streptococcus pneumoniae | Urine |
| 95 | streptococcus | Blood |
| 97 | Bordetella pertussis | Nasopharynx |
| CZ5 | Pseudomonas aeruginosa | Tracheal aspirate, blood culture |
| CZ6 | Serratia marcescen | Bronchoalveolar lavage |
| 46* | streptococcus | Bronchoalveolar lavage |
| 91* | Methicillin-resistant Staphylococcus | blood, bronchoalveolar lavage |
| CZ4* | streptococcus | Tracheal aspirate |

**Table S3. Comparison of class predictors for identifying influenza A infection. Genes present in more than one class predictor are highlighted in bold.**

| Study | Ramilo *et. al.* | Zaas *et. al.* | Parnell *et. al.* |
| --- | --- | --- | --- |
| Groups compared | Influenza A vs *E coli* or  *S pneumoniae* infection. | Symptomatic vs asymptomatic influenza A infection | H1N1 influenza A pneumonia vs bacterial pneumonia |
|  | APOBEC3C | RSAD2 | GINS2 |
|  | BST2 | IFI44L | RFC5 |
|  | C1orf29 | SIGLEC1 | UHRF1 |
|  | CD44 | LAMP3 | TYMS |
|  | cig5 | IFIT1 | FUT8 |
|  | dJ507I15.1 | **IFI44** | SLC2A5 |
|  | DNAPTP6 | SERPING1 | MCM6 |
|  | EEF1G | **IFI27** | STMN1 |
|  | EEF1G | ISG15 | RAD54L |
|  | EIF3S5 | **IFI44** | UBE2T |
|  | EIF3S7 | HERC5 | PCNA |
|  | EIF4B | LOC26010 | MCM4 |
|  | FLJ20035 | IFI6 | **IFI27** |
|  | FLJ38348 | LOC727996 | SQLE |
|  | G1P2 | IFIT3 | UAP1 |
|  | HADHA | OAS3 | TIMELESS |
|  | HSXIAPAF1 | OASL | ADK |
|  | **IFI27** | SEPT4 | BTG3 |
|  | IFI35 | XAF1 | GINS3 |
|  | **IFI44** | OAS1 | WEE1 |
|  | **IFI44** | **LY6E** | KCTD14 |
|  | KIAA0152 | MS4A4A | C6orf125 |
|  | **LY6E** | SIGLEC1 | FEN1 |
|  | **MX1** | TNFAIP6 | OIP5 |
|  | **OAS1** | CCL2 | PDE9A |
|  | PCBP2 | **OAS1** | C1orf135 |
|  | PFDN5 | **MX1** | MCM10 |
|  | PHACTR2 | TNFAIP6 | CBL |
|  | QARS | RTP4 | WFDC2 |
|  | RPL31 | OASL |  |
|  | RPL4 |  |  |
|  | SON |  |  |
|  | TRIM14 |  |  |
|  | USP18 |  |  |
|  | ZBP1 |  |  |


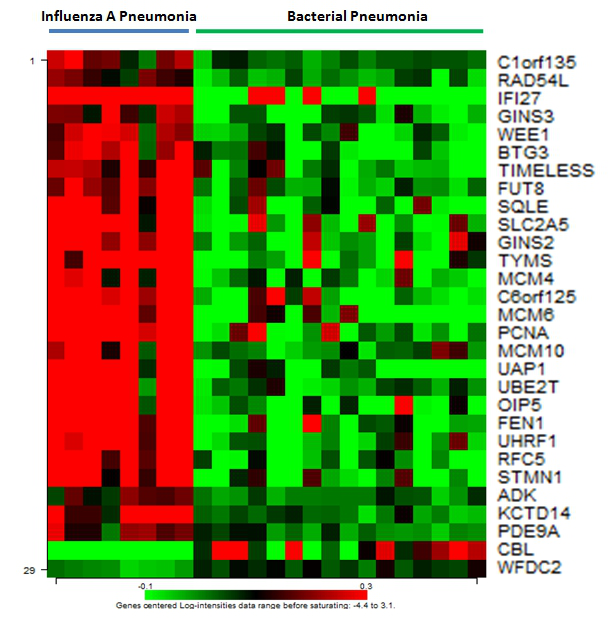


**Figure S1.** 29 Gene Classifier for day 1 samples of bacterial and H1N1 influenza A pneumonia patients

**
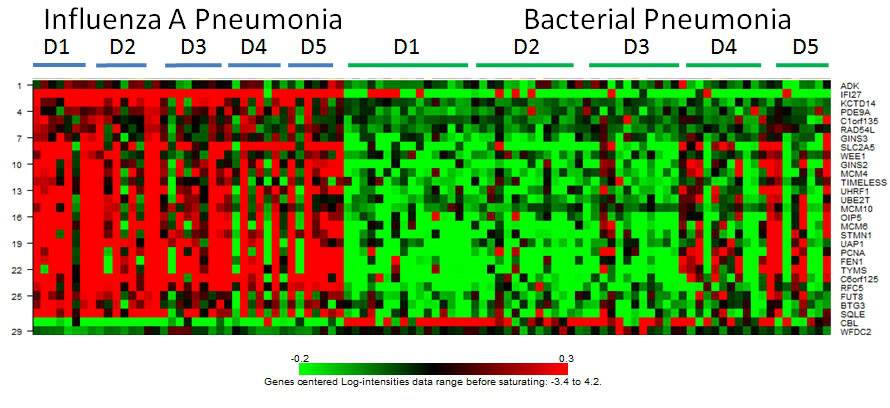
**

**Figure S2.** 29 gene classifier across 5 days for bacterial and H1N1 influenza A pneumonia patients
